# Inflammatory Mechanisms Contributing to Endothelial Dysfunction

**DOI:** 10.3390/biomedicines9070781

**Published:** 2021-07-06

**Authors:** Panagiotis Theofilis, Marios Sagris, Evangelos Oikonomou, Alexios S. Antonopoulos, Gerasimos Siasos, Costas Tsioufis, Dimitris Tousoulis

**Affiliations:** 1 1st Department of Cardiology, Hippokration General Hospital, University of Athens Medical School, 11527 Athens, Greece; panos.theofilis@hotmail.com (P.T.); masagris1919@gmail.com (M.S.); boikono@gmail.com (E.O.); alexios.antonopoulos@cardiov.ox.ac.uk (A.S.A.); ger_sias@hotmail.com (G.S.); ktsioufis@gmail.com (C.T.); 2 3rd Department of Cardiology, Thoracic Diseases General Hospital Sotiria, University of Athens Medical School, 11527 Athens, Greece

**Keywords:** endothelial dysfunction, inflammation, Nf-κB, adhesion molecules, selectins, NLRP3 inflammasome, shear stress

## Abstract

Maintenance of endothelial cell integrity is an important component of human health and disease since the endothelium can perform various functions including regulation of vascular tone, control of hemostasis and thrombosis, cellular adhesion, smooth muscle cell proliferation, and vascular inflammation. Endothelial dysfunction is encompassed by complex pathophysiology that is based on endothelial nitric oxide synthase uncoupling and endothelial activation following stimulation from various inflammatory mediators (molecular patterns, oxidized lipoproteins, cytokines). The downstream signaling via nuclear factor-κB leads to overexpression of adhesion molecules, selectins, and chemokines that facilitate leukocyte adhesion, rolling, and transmigration to the subendothelial space. Moreover, oscillatory shear stress leads to pro-inflammatory endothelial activation with increased monocyte adhesion and endothelial cell apoptosis, an effect that is dependent on multiple pathways and flow-sensitive microRNA regulation. Moreover, the role of neutrophil extracellular traps and NLRP3 inflammasome as inflammatory mechanisms contributing to endothelial dysfunction has recently been unveiled and is under further investigation. Consequently, and following their activation, injured endothelial cells release inflammatory mediators and enter a pro-thrombotic state through activation of coagulation pathways, downregulation of thrombomodulin, and an increase in platelet adhesion and aggregation owing to the action of von-Willebrand factor, ultimately promoting atherosclerosis progression.

## 1. Introduction

Cardiovascular diseases represent the primary cause of morbidity and mortality in western societies despite the breakthroughs in their diagnosis, treatment, and prevention. Several risk factors are implicated in their pathogenesis, such as arterial hypertension, diabetes mellitus (DM), smoking, and obesity. Interestingly, most of these processes are linked with endothelial dysfunction, the initial step of atherogenesis, which has been proven to be a precursor of adverse cardiovascular outcomes [1,2,3,4]. Recently, a lot of interest has been shown on the pro-inflammatory state stemming from the cluster of comorbidities frequently encompassing patients with cardiovascular diseases and its deleterious effect on atherosclerosis. Therefore, in the context of this narrative review, we present the inflammatory mechanisms involved in the development of endothelial dysfunction and the potential therapeutic implications according to the latest preclinical and clinical studies.

## 2. Physiology of the Vascular Endothelium

### 2.1. Endothelial Cell Anatomy and Function

The endothelium is an abundant organ consisting of a squamous cell monolayer that lines blood vessels, being in contact with the flowing blood. It consists of polarized endothelial cells (EC) adjacent to a basal lamina, together forming the tunica intima of blood vessels. ECs are frequently described as thin and slightly elongated with average dimensions of 30–50 μm length, 10–30 μm width, and 0.1–1 μm height. They are positioned along the vessel axis to mitigate the shear stress (SS) deriving from the blood flow. Even though once believed to be just a bystander, the endothelium has now been established as an endocrine organ, regulating the exchange of fluids, nutrients, and metabolites, and is characterized as a crucial mediator of various functions. Among their well-described properties is vascular tone regulation via vasoconstriction or relaxation, vascular remodeling, control of hemostasis and thrombosis, cellular adhesion, smooth muscle cell proliferation, and vascular inflammation as long as ECs remain in a healthy state [5,6]. 

#### 2.1.1. Regulation of Vascular Tone

The discovery of prostacyclin and its endothelial-related synthesis along with the work from Furchgott and Zawadzki concerning endothelial nitric oxide (eNO) demonstrated the importance of endothelium in vascular relaxation [7,8,9]. Consequently, tissue oxygen supply is dependent upon synthesis and release of NO, endothelial-derived hyperpolarizing factor (EDHF), arachidonic acid metabolites signaling via cyclooxygenase, lipoxygenase, and cytochrome P450 while the role of various molecules (angiotensin II (ATII), endothelin, urotensin, C-type natriuretic peptide, bradykinin, adrenomedullin, adenosine, purines, reactive oxygen species (ROS)) is vital in achieving the balance in vascular tone [10].

#### 2.1.2. The Role of Nitric Oxide

Nitric oxide is a molecule with pleiotropic functions in endothelial function. It is synthesized from L-arginine in endothelial cells, with calcium-calmodulin-dependent NO synthase (NOS) acting as a catalyst for this reaction. Three different subtypes of NOS (neuronal NOS (nNOS), inducible NOS (iNOS), and endothelial NOS (eNOS)) have been reported, each having different structural and functional characteristics. Calcium-dependent enzymes nNOS and eNOS are the constitutive NOS while iNOS is induced by immunological stimuli. Among the known required NOS co-factors are oxygen, nicotinamide adenine dinucleotide phosphate (NADPH), and tetrahydrobiopterin (BH_4_) [11]. Following its synthesis, it is distributed from the endothelial cell membrane to vascular smooth muscle cells leading to the activation of guanylate cyclase, which then converts GTP to cGMP, ultimately resulting in the removal of calcium and consequent relaxation of cells [12].

Other than its role in mediating the vascular tone via relaxation of smooth muscle cells, NO has an antithrombotic role due to the attenuation of platelet activation and aggregation, regulates the migration and adhesion of leukocytes on EC, and inhibits vascular smooth muscle cell proliferation [11]. Moreover, it has been implicated in the maintenance of endothelial integrity and permeability [13]. It should also be noted that NO is a potent oxygen free radical scavenger by enhanced decomposition of peroxynitrite to nitrate and nitrite and inhibition of neutrophil-related O^−^_2_ production via limiting NADPH oxidase activity [14,15].

## 3. Assessment of Endothelial Function

Several methods aiming at estimating endothelial function have been investigated and validated during the past decades. The main objective stemming from those examinations is the demonstration of vasodilation following a stimulus, either that being a vasodilating substance such as acetylcholine or reactive hyperemia-induced high shear stress, leading to an increase in NO and other endothelium-derived factors [16,17].

Invasive methods of endothelial function assessment are applied in the clinically relevant coronary circulation. In the setting of coronary angiography or intravascular ultrasound, the healthy endothelium leads to vessel dilation following administration of acetylcholine or other endothelial vasodilators whereas in endothelial dysfunction a lower vasodilatory response or even vasoconstriction might be observed. On the level of coronary microvasculature, alterations between baseline and coronary blood flow following stimulation with adenosine define coronary flow reserve (CFR) which is, however, affected by both endothelium and non-endothelium dependent mechanisms [6]. Even though these methods are considered the gold standard, their invasive nature and increased cost limit their widespread application and are only employed in patients requiring cardiac catheterization for other indications. Venous occlusion plethysmography is a semi-invasive approach via the unilateral cannulation of the brachial artery followed by administration of vasoactive agents and subsequent quantification of endothelium-dependent and independent vessel dilation, with the contralateral arm serving as a control [18].

The most frequently used method of endothelial function assessment has undoubtedly been flow-mediated dilation (FMD) of the brachial artery. After occlusion of the brachial artery via a blood pressure cuff inflated usually 50 mmHg over the systolic blood pressure of the participant for 5 minutes, reactive hyperemia ensues leading to an increase in endothelial shear stress which is a known NO stimulus [19]. Even though it is easily accessible, cost-effective, and correlates adequately with invasive measurements, the existence of various protocols (cuff position, duration-magnitude of occlusion, time stamps for post-deflation measurements), as well as significant inter- and intra-observer variability, are important limitations [19,20]. Over time, changes in FMD may add prognostic information regarding future cardiovascular risk [21,22]. Moreover, FMD has been correlated with several biomarkers of atherosclerosis and heart failure [23,24].

Finger plethysmography is another non-invasive method of estimating endothelial function [25]. It is based on the recording of finger arterial pulse amplitude by pneumatic probes after reactive hyperemia induction similarly to the FMD technique and the index between the examined and the control arm is calculated. However vascular dilatation is partially NO-dependent using this method [26], and it has been reportedly associated with coronary microvascular function but not with brachial artery FMD [27,28,29]. Lastly, retinal endothelial function assessment via provocation with flicker light has been recently proposed since vasoreaction is also partially dependent on NO. Although knowledge is still limited and no standardization protocols have been established [30], the retinal endothelial function has been linked with various diseases including diabetes mellitus [31], obesity [32], heart failure [33], and hypertension [34].

With regards to biomarkers of endothelial dysfunction, adhesion molecules, selectins, and prothrombotic molecules have been used in the past for the assessment of endothelial function, correlating well with traditional cardiovascular risk factors and incident cardiovascular risk despite having important drawbacks. Lately, interest has been shifted towards endothelial microparticles as a more specific means of endothelial function estimation, while studies have suggested a predictive value towards cardiovascular risk [35]. Endocan has also been described as an endothelial-specific marker that could be employed in disease states characterized by a dysfunctional endothelium [36].

## 4. Pathophysiology of Endothelial Dysfunction

### 4.1. eNOS Uncoupling

Endothelial dysfunction is the result of an imbalance between vasodilators and vasoconstrictors produced by endothelial cells, leading to an atheroprone phenotype consisting of vasoconstriction, leukocyte trafficking, inflammation, and coagulation-thrombosis. Reduced synthesis and availability of NO, due to impaired eNOS expression and activity, is frequently the initial mediator of endothelial dysfunction. In conditions of increased oxidative stress, the insufficient concentration of co-factors drives the production of superoxide instead of NO, a process known as NO uncoupling, with harmful downstream effects such as the generation of the pro-oxidant peroxynitrite which promotes mitochondrial and endothelial cell dysfunction [37,38].

The low bioavailability of the required co-factor BH_4_ has been proven to be the major determinant of NO uncoupling due to ROS-induced oxidation to BH_2_ [39]. Moreover, the inability of a salvage pathway of BH_4_ production, by recycling of oxidized BH_2_ to BH_4_ through the action of dihydrofolate reductase (DHFR), has also been reported through depletion of DHFR enzyme [40,41]. The role of NADPH oxidase in the reduced BH_4_ bioavailability has been long established [41,42]. 

Asymmetric dimethylarginine (ADMA) is a known endogenous inhibitor of NO formation through competition with L-arginine at the active NOS site [43]. Its synthesis is based on the proteolysis of methylated arginine, after its modification by protein-arginine methyltransferases [44]. Studies have also reported associations of endothelial ADMA with oxidative stress and superoxide production, highlighting its role in endothelial dysfunction [45]. Moreover, ADMA-induced oxidative stress could be the result of depleted BH_4_ stores, further leading to eNOS uncoupling and enhanced superoxide production. Last but not least, a pro-inflammatory role of ADMA has also been described [46], owing to leukocyte adhesion and the production of inflammatory mediators [47].

### 4.2. Cardiovascular Risk Factors and Endothelial Dysfunction

#### 4.2.1. Smoking

Tobacco and electronic cigarette smoking have been repetitively proven to adversely affect endothelial function by promoting oxidative stress [48,49]. The role of cyclooxygenases (COX), particularly COX-1, in endothelial activation following cigarette smoking has been also demonstrated [50]. Additionally, cigarette smoke constituents have been related to BH_4_ depletion and eNOS uncoupling, which promote endothelial dysfunction [51]. However, these deleterious actions on vascular endothelium appear to be partially reversible after cessation of smoking [52,53,54].

#### 4.2.2. Diabetes Mellitus

Diabetes mellitus, a global pandemic with increasing incidence and disastrous vascular complications [55], has been implicated in endothelial dysfunction. Hyperglycemia leads to disturbed NO bioavailability and ROS production, with overproduction of advanced glycation end products (AGEs), overexpression of their receptors (RAGE), and hexosamine pathway activation being also involved [56]. Indeed, initial preclinical studies have pointed to oxidative stress as the earliest abnormality in diabetes mellitus natural history [57]. 

#### 4.2.3. Arterial Hypertension

Moving on to arterial hypertension, its deleterious effect on endothelial function has been consistently proven in animal as well as human studies [58], while a prognostic role of endothelial dysfunction in hypertensives has also been documented [59]. An increase in ROS has been the principal finding in studies evaluating possible pathophysiologic mechanisms that include increased expression of NADPH oxidase (NOX) [60], ADMA [61], endothelin-1 [62], and angiotensin-II [63].

#### 4.2.4. Hypercholesterolemia

Concluding with hypercholesterolemia, the role of oxidized low-density lipoprotein (oxLDL) is crucial in the development of endothelial dysfunction. High amounts of oxLDL lead to an imbalance of eNOS and iNOS mediated by High-mobility group box 1 (HMGB1)-toll-like receptor (TLR) pathway and lectin-type oxLDL receptor 1 (LOX-1)-nuclear factor-κB (NF-κB) pathway respectively, ultimately resulting in endothelial dysfunction due to EC apoptosis and reduced protective autophagy [64]. Even though oxLDL impairs endothelial function, this effect appears to be reversible via the action of high-density lipoprotein [65]. Lipoprotein(a) has recently emerged as a marker of increased cardiovascular risk, with preliminary experimental data reporting adverse endothelial effects via the activation of monocytes [66]. However, further investigation is required to elucidate its role in endothelial function.

## 5. The Role of Inflammation in Endothelial Dysfunction

**Figure 1:**
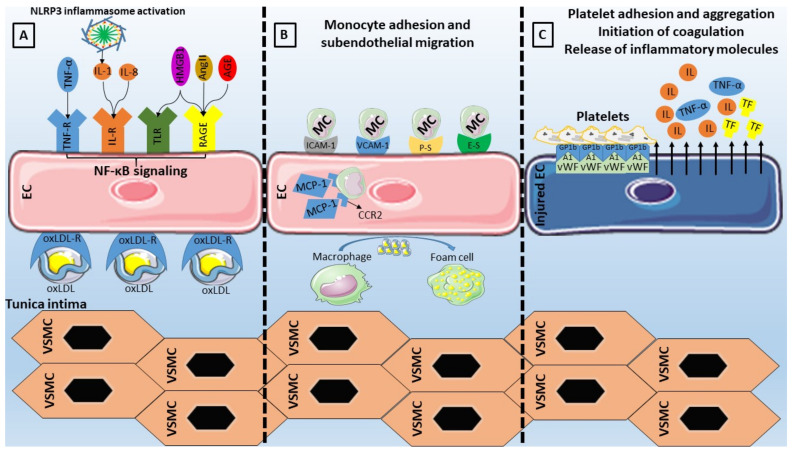
Inflammatory activation of endothelial cells (ECs). (**A**) Stimulation of EC receptors by damage-associated molecular patterns (High mobility group box 1 (HMGB1)), inflammatory cytokines (tumor necrosis factor (TNF)-α, interleukins (ILs)), oxidized low-density lipoproteins (oxLDL), advanced glycation end products (AGEs), and angiotensin (Ang)-II promotes nuclear factor-κB (NF-κB) signaling which results in (**B**) upregulation of adhesion molecules (vascular cell adhesion molecule (VCAM)-1, intercellular adhesion molecule (ICAM)-1, E-Selectin (E-S), P-Selectin (P-S)) with subsequent monocyte (MC) adhesion and subendothelial transmigration with the aid of monocyte chemoattractant protein (MCP)-1 and its receptor C-C chemokine receptor type 2 (CCR2). Monocytes proceed to differentiate into macrophages that phagocytose oxLDL to become foam cells. (**C**) Injured endothelial cells release inflammatory mediators and tissue factor (TF) further promoting inflammation and coagulation, while the release of von-Willebrand factor (vWF) from the Weibel-Palade bodies results in platelet adhesion and aggregation following the binding with platelet glycoprotein (GP)1b. NLRP3: NLR family pyrin domain containing 3, TLR: toll-like receptor, RAGE: receptor of advanced glycation end products, VSMC: vascular smooth muscle cell.

In cases of infections or tissue injury, endothelial cells undergo morphological and functional modifications. This process, termed endothelial activation, is triggered by various stimuli including bacterial endotoxins, inflammatory cytokines (tumor necrosis factor (TNF)-α, ILs, and interferon-γ), or pattern recognition receptor activation (PRR) following the identification of pathogen-associated molecular patterns (PAMPs) or damage-associated molecular patterns (DAMPs) [67,68]. Examples of such PRR with implications in endothelial dysfunction include toll-like receptors (TLRs) and NOD-like receptors (NLRs), among others [69]. Following activation of PRRs, upregulation of pro-inflammatory molecule expression leads to an increasing burden of sustained inflammation with local and systemic complications (Figure 1) [70].

### 5.1. TLRs and Endothelial Dysfunction

TLRs are important regulators of the immune system due to their pattern recognition and the ability to initiate inflammation. Two distinct pathways have been described upon activation of a TLR; the myeloid differentiation primary response protein 88 (MyD88)-dependent pathway involving the early phase NF-κB activation and the myD88-independent pathway associated with the late phase NF-κB activation [71].

HMGB1, a major non-histone protein that is overexpressed in the setting of DM [72], is one of the most well-studied DAMPs firmly correlating with chronic low-grade inflammation [73]. Its ability to bind with TLR4 and RAGE leads to activation of the immune system and the initiation of inflammation [74]. Moreover, increased trimethylamine N-oxide (TMAO) in the setting of gut dysbiosis was recently found to be related to endothelial cell dysfunction through an HMGB1-TLR4 pathway [75]. Arterial hypertension is another TLR4 stimulus owing to the action of angiotensin II as demonstrated in aortic samples of hypertensive rats [76]. Eventually, and following TLR4 activation, downstream inhibition of antioxidant enzymes, activation of NADPH oxidase, and upregulation of pro-inflammatory cytokines production lead to endothelial cell dysfunction.

### 5.2. NLRP3 Inflammasome and Endothelial Dysfunction

Inflammasomes, first mentioned nearly two decades ago, have been described as crucial in the innate immunity processes, implicated in inflammatory diseases’ natural history [77,78]. The most well-characterized member has undoubtedly been the NLRP3 inflammasome, with research showing associations with multiple diseases with deregulated inflammation and dysfunctional endothelium (DM, arterial hypertension, obesity, atherosclerosis) [79,80,81,82].

NLRP3 inflammasome activation can be the outcome of various signals, namely ion fluxes, mitochondrial dysfunction, and ROS overproduction [83], while noncanonical and alternative activation have also been proposed [84,85]. Interestingly the alternative NLRP3 inflammasome activation, involving the TLR4–TIR-domain-containing adaptor-inducing interferon-β (TRIF)–receptor-interacting serine/threonine-protein kinase 1 (RIPK1)–Fas-associated protein with death domain (FADD)–CASP8 signaling pathway, is not involved in pro-inflammatory cell death (pyroptosis) [85].

In chronic low-grade inflammatory diseases, such as cardiovascular diseases, an increased burden of ROS has been observed. ROS-induced inflammation via expression of inflammatory cytokines, including IL-1β, has been linked with NLRP3 inflammasome activation, which is essential for the proteolytic cleavage of those inflammatory mediators [86]. Available knowledge points towards autophagy and ROS as negative and positive regulators of NLRP3 inflammasome. Cardiovascular risk factors are also implicated, with obesity-induced inhibition of mitophagy and cholesterol crystal-induced inflammasome activation resulting in IL-1βand IL-18 production [87,88,89].

### 5.3. The Role of NF-κB and Adhesion Molecules

Upon exposure to conditions of increased stress, the inflammatory and pro-coagulant effects of endothelial cells are mediated by the NF-κB signaling [90], with downstream upregulation of target genes of adhesion molecules (VCAM-1, ICAM-1, E-selectin, P-Selectin) and chemokines (monocyte chemoattractant protein (MCP)-1) [91,92,93,94]. Following that, the adhesion and transmigration of inflammatory cells (monocytes, T-lymphocytes) occur, with the ensuing activation of neutrophils leading to overexpression of inflammatory cytokines, chemokines, growth factors, and ROS. Moreover, NF-κB is responsible for the release of inflammatory mediators that modulate smooth muscle cell activation, further contributing to inflammation and atherosclerosis progression [95].

Concerning adhesion molecules specifically (Table 1), VCAM-1 has been considered an essential component of the endothelial activation cascade as it is involved in the selective adhesiveness of monocytes and lymphocytes, as they have been found to express the counterreceptor very late antigen (VLA)-4 [96,97]. ICAM-1 is also implicated in the interaction between endothelial cells and monocytes through its ligands (lymphocyte function-associated antigen-1, macrophage-1 antigen), by enabling the adhesion and migration of leukocytes [98]. Moving on to selectins (P-Selectin, E-Selectin, and L-Selectin), all of which can be found on the surface of endothelial cells, leukocytes, and platelets, their structure is based on an N-terminal carbohydrate recognition domain, an epidermal growth factor-like domain, a varying number of short consensus repeats that have homology to complement regulatory domains (2, 6, and 9 within L-, E-, and P-selectin respectively), a transmembrane region, and a C-terminal cytoplasmatic tail [99]. The largest member of the family, P-Selectin, is situated on the membrane of the Weibel-Palade bodies of endothelial cells. The presence of its primary ligand (P-selectin glycoprotein ligand-1) on the surface of leukocytes indicates its role in leukocyte adhesion and rolling on endothelium, while available evidence points to an additional signaling role within the endothelium [99]. E-Selectin, whose upregulation is based on the NF-κB binding to regulatory domains in its promoter, has the capability of retarding leukocyte rolling leading to leukocyte arrest, making it pivotal in leukocyte trafficking and inflammatory responses [99]. The role of selectins in atherosclerotic diseases has been extensively investigated, with both molecules being present on endothelial cells of atherosclerotic plaques [100], while E-selectin or P-selectin deficient mice displayed attenuated atherosclerosis [101,102].Last but not least, MCP-1 has been the first reported inflammatory chemokine that is secreted from endothelial cells and monocytes, with its function consisting of leukocyte mobilization towards the subendothelium by binding on the CCR2 receptor, thus contributing to atherosclerosis [103]. Animal studies have shown that MCP-1 or CCR2 deficiency leads to lower lipid deposition and slowing of the atherosclerotic process [104,105], while its overexpression results in opposite effects with macrophage accumulation and atherosclerosis acceleration [106]. Moreover, another study has reported a role of MCP-1 on endothelial cell apoptosis [107]. Importantly, the role of adhesion molecules as markers of cardiovascular disease and incident cardiovascular risk has also been studied [108,109,110,111,112,113,114].

### 5.4. The Pro-Inflammatory Effect of NOX

NOX are the only family of enzymes primarily implicated in ROS generation, since other ROS regulators require an external ROS source to become involved in their formation. Therefore, their role in the initiation of the oxidative stress cascade is critical and depends upon the presence of cardiovascular risk factors. In such cases, NOX overproduction results in activation of inflammatory pathways together with eNOS uncoupling and scavenging of antioxidants [119].

From the four described NOX expressed in EC, NOX1, NOX2, and NOX5 are implicated in vascular diseases while an antiatherogenic role is speculated for NOX4 [119]. Even though NOX exert their pro-atherogenic effects via promotion of oxidative stress and impairment of NO bioavailability, recent reports have linked NOX-derived ROS to propagate NF-κB signaling and, consequently, the release of adhesion molecules and pro-inflammatory mediators [120]. Interestingly, exposure to oscillatory SS resulted in an upregulated expression of NOX2 via the action of sterol regulatory element binding protein 2, ultimately leading to NLRP3 inflammasome activation [121].

### 5.5. Neutrophil Extracellular Traps

Neutrophil extracellular traps (NETs), consisting of nuclear chromatin, histones, and proteins of various origins, have been recently attached to the inflammatory background of atherosclerotic cardiovascular diseases [122]. Excessive NET production may result in vascular leakage and endothelial-to-mesenchymal transition by degradation of VE-cadherin [123], as well as in complement activation leading to endothelial injury [124].

Several pro-atherosclerotic conditions have been characterized by NETosis including hyperglycemia, dyslipidemia, and obesity. Starting with hyperglycemia, it has been proven that NET production is highly NADPH oxidase-dependent in the setting of DM [125], eventually resulting in endothelial injury via damage to the endothelial glycocalyx [126]. In cases of dyslipidemia, oxLDL may act on neutrophils to enhance NET formation, with the resulting product being able to propagate endothelial dysfunction [127]. Obesity is another condition with presumed NETosis owing to its pro-inflammatory actions and, as demonstrated in diet-induced obesity mouse models, the NET formation was assumed to be the orchestrator of endothelial dysfunction [128]. Studies assessing the association of NETs with endothelial function in humans are scarce, however, highlighting the continuous research that ought to be performed in this field.

### 5.6. Shear Stress

**Figure 2:**
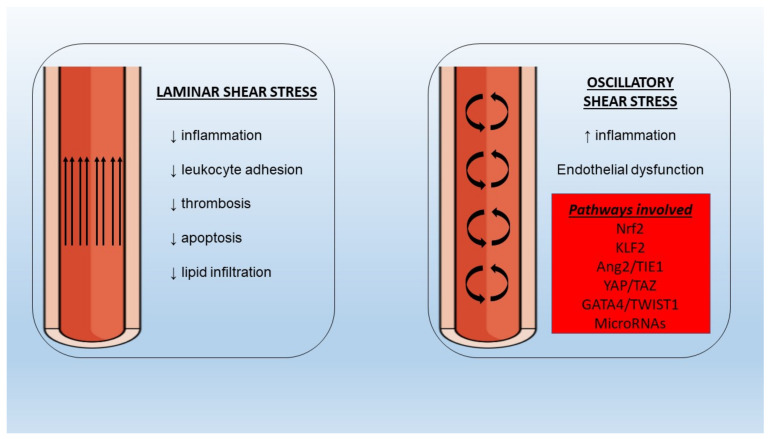
Different properties of laminar and oscillatory shear stress under normal and pathologic conditions with implications for inflammation and endothelial dysfunction. Nrf2: nuclear factor erythroid 2–related factor 2, KLF2: Krüppel-like factor-2, Ang2: angiopoietin-2, TIE1: tyrosine kinase with immunoglobulin-like and EGF-like domains-1, YAP: yes-associated protein, TWIST1: twist-related protein-1.

Endothelial cell regulation at the mechanical level is achieved via SS forces. In the setting of a laminar SS the beneficial, atheroprotective functions of endothelium remain intact (Figure 2). However, in areas of bifurcations, there is a differentiation of flow pattern towards turbulence, with an altered phenotype leading to increased adhesion of monocytes, proliferation, and apoptosis. It has been shown that the application of SS leads to the activation of multiple cell membrane mechanosensors such as integrins [129], G protein-coupled receptors [130], and endothelial glycocalyx [131], among others, with ensuing activation of downstream signaling pathways associated with functional gene expression. It is important to note that laminar SS application on cultured endothelial cells resulted in enhanced expression of eNOS [132], with Akt phosphorylation and subsequent eNOS phosphorylation at Ser 1177 being important for NO production [133].

In the presence of oscillating or low SS an increased burden of NF-κB molecules has been observed [134], pointing to a pro-inflammatory effect on the underlying endothelium which is potentially mediated by nuclear factor erythroid 2–related factor 2 (Nrf2) and Krüppel-like factor (KLF)-2 [135,136]. Moreover, low SS could be responsible for the disturbances of angiopoietin (Ang)/tyrosine kinase with immunoglobulin-like and EGF-like domains 1 (TIE1) axis via upregulation of the pro-inflammatory Ang2 in endothelial cells [137]. Recent evidence from omics studies of endothelial cells has proposed additional novel links between SS and genes involved in embryonic development. To begin with, the yes-associated protein (YAP)/TAZ pathway promotes JUN N-terminal kinase-mediated inflammation in conditions of low SS [138,139]. Twist-related protein 1 (TWIST1) is another molecule involved in developmental processes that has been investigated since it was found increased in aortic areas of low SS [140]. In a preclinical study, TWIST1 was induced in a GATA4-dependent manner after exposure to low SS with inflammation and endothelial-to-mesenchymal transition processes being also promoted, thus leading to endothelial dysfunction [141]. Several other pathways (bone morphogenetic protein signaling, WNT signaling) have been implicated in the interaction between shear stress, inflammation, and endothelial function and merit further validation in animal studies [142,143].

Flow alterations have important implications in microRNA regulation. MicroRNAs are small non-coding RNA molecules consisting of 18–24 nucleotides that contribute to the regulation of numerous cellular functions such as multiplication, differentiation, apoptosis, and death, especially in situations of inflammation or trauma, via RNA interference and post-translational modification in gene expression. It is, therefore, expected that deregulated expression of epigenetics will affect inflammatory pathways and endothelial cell function. Starting with microRNA-126, which is believed to be a key regulator of vascular endothelial homeostasis and inflammation [144,145], even though early studies suggested a pro-atherogenic role, the latest evidence proposes an anti-inflammatory, endothelial-protective mechanism of action which is lost in conditions of disturbed flow [146,147,148]. MicroRNA 19a, a member of the microRNA-17~92a, is overexpressed in low SS and exerts pro-inflammatory effects by targeting HMGB1 [149]. MicroRNA-92 from the same cluster is also overexpressed in disturbed flow conditions and targets KLF2, KLF4, and the suppressor of cytokine signaling 5 (SOCS5), leading to endothelial inflammation and dysfunction [150]. Next, microRNA-663 overexpression under low SS conditions appears to induce endothelial inflammation with multiple targets involved [151]. MicroRNA-712, solely found in murine animal models, and its human homolog microRNA-205 are upregulated in situations of disturbed flow and are involved in endothelial cell inflammation mediated by the loss of tissue inhibitor of metalloproteinase-3 (TIMP3) expression [152]. MicroRNA-181 is also reduced under low SS conditions leading to NLR family pyrin domain containing 3 (NLRP3) inflammasome-dependent pyroptosis of endothelial cells [153].

### 5.7. Endothelial Dysfunction in Chronic Inflammatory Diseases

An excess of adverse cardiovascular events in individuals with chronic autoimmune inflammatory diseases has been noted, with endothelial dysfunction potentially being the crucial link. Since these conditions are characterized by a significant inflammatory burden, the overexpression of inflammatory cytokines leads to increased oxidative stress and dyslipidemia, while the role of autoantibodies in the development of endothelial dysfunction remains to be elucidated [154].

Starting with rheumatoid arthritis, the presence of endothelial dysfunction is frequently present, as shown in a recent meta-analysis [155]. However, the correlations with disease duration, activity, and remission have been inconsistent [156]. In the case of systemic lupus erythematosus, which is also accompanied by increased cardiovascular disease rates [157], a higher prevalence of endothelial dysfunction has been observed the latest meta-analysis [158]. The presence of endothelial dysfunction has been described in patients with psoriasis, possibly due to the reduced NO bioavailability, which is correlated to symptom severity [159]. Importantly, the presence of impaired coronary endothelial function, assessed by positron emission tomography, in the above-mentioned inflammatory states was related to an increased rate of major adverse cardiovascular events [160]. Lastly, impaired endothelial function has been documented in patients with inflammatory bowel disease, correlated with disease activity in the majority of studies [161].

## 6. A Link between Inflammation and Thrombosis in Endothelial Dysfunction

Under healthy situations, the endothelium is capable of producing inhibitors of thrombin synthesis and activity, therefore maintaining the balance between coagulation and fibrinolysis. However, in situations of vascular injury and inflammation, the ensuing endothelial dysfunction and EC activation lead to a pro-thrombotic phenotype via the upregulation of coagulation factors. Activated EC can express active tissue factor on the cell surface thereby initiating the extrinsic pathway of coagulation [162], with experimental evidence highlighting the role of inflammatory mediators in this process, including TNF-α [163], CD40 ligand [164], and other inflammatory cytokines [165]. The role of endothelial activation in the intrinsic pathway of coagulation is less clearly understood, with a recent report demonstrating the possible effect of inflammation on factor IX production following treatment of human umbilical vein endothelial cells with TNF [166].

Thrombomodulin is another mediator of thrombosis that is highly expressed in EC surface [167]. Its anti-thrombotic mechanism of action revolves around binding to thrombin, upregulation of activated protein C, and catalyzing thrombin inhibition by antithrombin [168,169]. In a recent study by Yang et al., Nur77 and Nor1 were identified as potential regulators of thrombomodulin expression [170] while inflammatory stimuli (CRP, oxLDL) have also been associated with downregulated expression and, thus, pro-thrombotic effects [171,172]. 

Activation of endothelial cells also leads to platelet mobilization. In physiologic conditions, the vascular endothelium is responsible for inhibition of platelet aggregation and adhesion through the release of NO and prostaglandins among others. However, in conditions of endothelial activation and consequent vascular injury, there is a release of various mediators including von-Willebrand factor (vWF), P-selectin, and angiopoietin-2 from the Weibel-Palade bodies of endothelial cells. Regarding vWF, a crucial molecule associated with platelet activation and coagulation, it was recently shown that endothelial-derived vWF was responsible for thrombus formation while platelet-derived vWF had only minor contributions [173], with similar findings observed concerning the vWF-dependent atherosclerotic process [174]. Inflammatory stimuli such as cytokines and superoxide anions lead to an increase in vWF [175,176], which is also considered an acute phase reactant. Therefore, it has a dual role in thrombosis via the facilitation of platelet adhesion, aggregation, and prevention of factor VIII proteolytic degradation as well as in inflammation through leukocyte mobilization, complement activation, and NETosis [177].

Thrombosis as a result of endothelial dysfunction is also prevalent in severe bacterial and viral infections. In septic conditions, the increased concentration of inflammatory cytokines, reactive oxygen species, and NETs leads to a prothrombotic endothelial state and vascular leakage [178]. Consequently, the incident immunothrombosis may result in life-threatening sepsis-associated disseminated intravascular coagulation [179]. Immunothrombosis is especially relevant in the context of the ongoing coronavirus disease 2019 pandemic, where the endotheliitis from severe acute respiratory syndrome coronavirus 2 has been associated with an increased risk of thrombotic complications [180].

## 7. Clinical Implications and Future Directions

It is evident that despite optimal control of cardiovascular risk factors, a significant burden of cardiovascular mortality exists, with endothelial dysfunction being an important prognostic tool for incident adverse cardiovascular events [181,182]. As demonstrated in a study of patients with coronary artery disease (CAD) who underwent noninvasive evaluation of endothelial function with FMD of brachial artery, a significantly higher number of events was noted in those with consistently impaired endothelial function despite optimal medical therapy. Lifestyle modifications are the initial step towards the amelioration of endothelial function, as observed in several studies of exercise [183,184], diet [185], and smoking cessation [186].

Concerning pharmacologic treatment, previous research has focused on evaluating the changes in endothelial function after treatment with statins. Their pleiotropic mechanism of action (anti-inflammatory, antioxidant) appears to improve endothelial dysfunction as observed in numerous studies [187,188], independently from their lipid-lowering property [189]. Several other agents have been investigated (sodium-glucose cotransporter-2 inhibitors, glucagon-like peptide-1 receptor agonists, colchicine), albeit less extensively, with encouraging results according to the available preclinical and clinical evidence [190,191,192,193,194].

With a look to the future, it seems inevitable that precision medicine will become the mainstay of medical treatment since genetic and epigenetic variations between individuals could be the crucial contributing factor in atherosclerosis development. Studies have already evaluated the impact of such abnormalities in gene expression on dysfunctional endothelial phenotypes [195,196,197,198]. Furthermore, epigenetic manipulation via microRNA mimics or inhibitors is another appealing intervention under rigorous investigation in various diseases. In the context of inflammation and endothelial dysfunction, several microRNAs have been studied based on their action on various pathways (Table 2) and could end up being valuable diagnostic and therapeutic in the years to come.

## 8. Conclusions

Endothelial dysfunction is frequently mentioned in the initial steps of atherogenesis, promoted by the highly prevalent cardiovascular risk factors. Inflammation plays an important role in its development, with recent studies providing additional knowledge on the complex pathophysiology of endothelial dysfunction. Amelioration of endothelial function could represent an additional step in cardiovascular risk reduction at the early stages of atherogenesis, with the role of genetic and epigenetic manipulation being under rigorous investigation.

## Figures and Tables

**Table 1:** Clinical implications of adhesion molecules involved in the interplay between endothelial dysfunction and inflammation.

Adhesion Molecule	Ligand	Role	Clinical Significance
ICAM-1	LFA-1 Mac-1	Leukocyte adhesion	ICAM-1 correlates with the incidence of CAD and carotid atherosclerosis independently of known cardiovascular risk factors [115].
VCAM-1	VLA-4	Leukocyte adhesion	Baseline VCAM-1 is increased in initially healthy middle-aged men who develop cardiovascular disease [116].
E-Selectin	ESL PSGL-1	Leukocyte adhesion	E-Selectin correlates with the incidence of CAD and carotid atherosclerosis independently of known cardiovascular risk factors [115].
P-Selectin	PSGL-1	Leukocyte adhesion	Elevated P-selectin levels predict early adverse events in patients with presumed CAD [117].
MCP-1	CCR2	Monocyte chemotaxis	Association of MCP-1 with risk of incident PAD and CAD independently of traditional cardiovascular risk factors [118].

ICAM-1: Intercellular adhesion molecule-1, VCAM-1: vascular cell adhesion molecule-1, MCP-1: monocyte chemoattractant protein-1, LFA-1: leukocyte function-associated antigen-1, VLA-4: very late antigen-4, ESL: E-selectin ligand, PSGL-1: P-selectin glycoprotein ligand-1, CCR2: C-C chemokine receptor type 2, CAD: coronary artery disease, PAD: peripheral arterial disease.

**Table 2:** Favorable effects of microRNA modulation on endothelial dysfunction and inflammation.

MicroRNA	Intervention	Target	Endothelial Effect
302c-3p [199]	↑	NLRP3 inflammasome	↓ EC pyroptosis
1929-3p [200]	↑	endothelin A receptor, NLRP3 inflammasome	↓ EC injury and vascular remodeling
181b-5p [153]	↑	STAT3/NLRP3 inflammasome	↓ EC pyroptosis
495 [201]	↑	NLRP3 inflammasome	↓ EC inflammation, apoptosis and ↑ EC proliferation
20b [202]	↑	TXNIP/NLRP3	↑ EC viability
520c-3p [203]	↑	NF-κB/Akt pathway	↓ EC apoptosis
216a [204]	↓	Smad7	↓ EC adhesive ability to monocytes
21 [205]	↓	Smad7	↓ endothelial-to-mesenchymal transition
17–3p [206]	↑	NIK and IKKβ binding protein	↓ monocyte adhesion to EC
217 [207]	↑	Early growth response protein-1	Relieve of EC growth inhibition, ↓ endothelial inflammation
200a [208]	↑	EZH2-Mediated Methylation of STAT3	↓ EC injury, apoptosis, and inflammation
200a [209]	↑	KEAP1/NRF2	↓ oxidative stress, inflammation, and endothelial dysfunction
383 [210]	↓	Sirtuin 1	↓ EC apoptosis and ROS production
34a [211]	↓	Sirtuin 1	↓ EC inflammation, oxidative stress, and endothelial dysfunction
34a [212]	↓	Sirtuin 1	Preservation of endothelium-dependent vasorelaxation
204 [213]	↓	Sirtuin 1	Mitigation of EC dysfunction
181a/181b [214]	↑	TAB2, NEMO	↓ adhesion molecules expression and monocyte-EC interaction
200a/200b [215]	↑	O-linked N-acetylglucosamine transferase	↓ EC inflammation and monocyte adhesion to EC

NLRP3: NLR family pyrin domain containing 3, EC: endothelial cell, STAT3: signal transducer and activator of transcription 3, TXNIP: thioredoxin interacting protein, NF-κB: nuclear factor kappa-light-chain-enhancer of activated B cells, Akt: protein kinase B, Smad7: SMAD family member 7, IκBα: inhibitor of kappa Bα, EZH2: enhancer of zeste homolog 2, KEAP1:Kelch-like ECH-associated protein 1, NRF2: nuclear factor erythroid 2-related factor 2, ROS: reactive oxygen species, NEMO: NF-kB essential modulator. ↑ indicates agonism, ↓ indicates antagonism.
